# Detection of Experimental Colorectal Peritoneal Metastases by a Novel PDGFRβ-Targeting Nanobody

**DOI:** 10.3390/cancers14184348

**Published:** 2022-09-06

**Authors:** Esther Strating, Sjoerd Elias, Guus van Scharrenburg, Kaisa Luoto, André Verheem, Inne Borel Rinkes, Herman Steen, Onno Kranenburg

**Affiliations:** 1 Laboratory Translational Oncology, Division Imaging and Cancer, University Medical Center Utrecht, Heidelberglaan 100, 3584 CX Utrecht, The Netherlands; 2 Julius Center for Health Sciences and Primary Care, University Medical Center Utrecht, Utrecht University, Heidelberglaan 100, 3584 CX Utrecht, The Netherlands; 3 BiOrion Technologies BV, MediTech Center, L.J. Zielstraweg 1, 9713 GX Groningen, The Netherlands

**Keywords:** platelet-derived growth factor receptor beta, colorectal cancer, peritoneal metastases, molecular imaging

## Abstract

**Simple Summary:**

Colorectal cancer can metastasize to multiple distant sites. Metastases growing within the peritoneal cavity cause a high degree of morbidity and are associated with very poor survival. Moreover, peritoneal metastases are difficult to detect using conventional imaging methods. Consequently, peritoneal metastases are generally under-diagnosed and their response to therapy is difficult to assess. An extensive molecular and cellular analysis of colorectal peritoneal metastases revealed that these lesions express very high levels of specific markers that could serve as targets for imaging-based diagnosis and treatment. In the present report, we explore the potential value of one such marker, PDGFRB, to serve as a target for peritoneal metastasis detection by molecular imaging. Therefore, we generated a PDGFRB-binding llama nanobody and demonstrate its utility in detecting peritoneal metastases in mice. The clinical development of PDGFRB-targeting tracers may help to improve the diagnosis of peritoneal metastases and the clinical management of this highly aggressive disease entity.

**Abstract:**

Peritoneal metastases in colorectal cancer (CRC) belong to Consensus Molecular Subtype 4 (CMS4) and are associated with poor prognosis. Conventional imaging modalities, such as Computed Tomography (CT) and Fluorodeoxyglucose-Positron Emission Tomography (FDG-PET), perform very poorly in the detection of peritoneal metastases. However, the stroma-rich nature of these lesions provides a basis for developing molecular imaging strategies. In this study, conducted from 2019 to 2021, we aimed to generate a Platelet-Derived Growth Factor Receptor beta (PDGFRB)-binding molecular imaging tracer for the detection of CMS4 CRC, including peritoneal metastases. The expression of PDGFRB mRNA discriminated CMS4 from CMS1-3 (AUROC = 0.86 (95% CI 0.85–0.88)) and was associated with poor relapse-free survival. PDGFRB mRNA and protein levels were very high in all human peritoneal metastases examined (*n* = 66). Therefore, we generated a PDGFRB-targeting llama nanobody (VHH1E12). Biotin-labelled VHH1E12 bound to immobilized human and mouse PDGFRB with high affinity (EC50 human PDGFRB = 7 nM; EC50 murine PDGFRB = 0.8 nM), and to PDGFRB-expressing HEK293 cells grown in vitro. A pharmacokinetic analysis of IRDye-800CW-conjugated VHH1E12 in mice showed that the plasma half-life was 6 min. IRDye-800CW-conjugated VHH1E12 specifically accumulated in experimentally induced colorectal cancer peritoneal metastases in mice. A tissue analysis subsequently demonstrated co-localization of the nanobody with PDGFRB expression in the tumour stroma. Our results demonstrate the potential value of PDGFRB-targeted molecular imaging as a novel strategy for the non-invasive detection of CMS4 CRC, in particular, peritoneal metastases.

## 1. Introduction

Mortality from colorectal cancer (CRC) is virtually always the consequence of metastatic spread to distant sites in the body such as the liver, the peritoneal cavity, and the lungs [1] Peritoneal metastasis formation is associated with high morbidity and very poor prognosis [2,3]. When left untreated, the median overall survival of this patient group is approximately five months [4]. Moreover, the benefit of systemic chemotherapy is very low in patients with peritoneal metastases [2,5]. 

The diagnostic tools that are used for detecting peritoneal metastases include surgical exploration (laparoscopy) and conventional radiological imaging techniques, i.e., Computed Tomography (CT) and/or Fluorodeoxyglucose-Positron Emission Tomography (FDG-PET). However, laparoscopy is an invasive surgical procedure and leaves important intra- and extra-peritoneal regions unexamined. It can also lead to the seeding of abdominal wall metastases [6]. Moreover, CT and FDG-PET perform poorly due to the relatively small size of individual lesions and the limited contrast and spatial resolution [7,8]. Consequently, it is very difficult to detect peritoneal metastases and monitor their response to systemic or intraperitoneal treatment. Over the past decade, this has resulted in the active exclusion of patients with peritoneal metastases from clinical trials [9]. Recent studies indicate that diffusion-weighted Magnetic Resonance Imaging (DW-MRI) has a superior sensitivity and specificity in the detection of peritoneal metastases [10,11,12]. In addition, molecular imaging using Fibroblast Activation Protein (FAP) inhibitor (FAPI) as a PET tracer may be a promising alternative imaging modality for the detection of peritoneal metastases [13,14,15,16].

Recently, we performed an extensive molecular analysis of a cohort of colorectal peritoneal metastases and the primary tumours that give rise to them. We found that these tumours form a near-homogeneous entity of Consensus Molecular Subtype 4 (CMS4) [17,18,19], a subtype that is characterized by a high content of stromal myofibroblasts [20,21,22], which express high levels of FAP, but also Platelet-Derived Growth Factor Receptor alpha (PDGFRA) and Platelet-Derived Growth Factor Receptor beta (PDGFRB). Encouraged by the success of FAPI-PET [15,16], we set out to study the potential value of PDGFRB-targeted molecular imaging for the detection of peritoneal metastases and, potentially, other CMS4-like stroma-rich tumour types. 

## 2. Materials and Methods

### 2.1. Colorectal Cancer RNA Sequencing Datasets 

In this paper we made use of three colorectal cancer RNA sequencing datasets. The relation between PDGFRB expression and CMS classification was assessed using a composite cohort of 3232 primary CRC tumours [21]. Next, the TCGA dataset containing 592 primary tumours was compared to primary tumours from patients with peritoneal involvement (*n* = 12) and the peritoneal metastasis derived from them (*n* = 35) [17] (GSE190609).

### 2.2. CMS Classification

RNA sequencing samples were classified using the CMS classifier R package (https://github.com/Sage-Bionetworks/CMSclassifier, accessed on 1 June 2021), as previously described [21].

### 2.3. Animals

All animal experiments were conducted in accordance with the guidelines and legislation of the Central Commission of Animal Experiments (Centrale Commissie Dierproeven) in the Netherlands. This includes the in vivo studies in mice, as well as the immunization of llamas for the generation of the nanobody. 

### 2.4. VHH Production

Two llamas were immunized twice with pre-treated recombinant human PDGFRB extracellular domain His-C, produced by U-protein Express BV, Utrecht, The Netherlands. PDGFRB was pretreated with an excess of human PDGF-BB to prevent the development of nanobodies that are directed against active dimerization regions or PDGF-BB binding regions. The rest of the production protocol was performed by QVQ Holding BV, Utrecht, The Netherlands. After the immunization, PBMCs were collected from which total RNA was isolated. The total RNA was used to create phage-display libraries. RNA was transcribed into cDNA using a reverse transcriptase Kit (Invitrogen, Waltham, MA, USA). Both VHH fragments as well as heavy chain fragments (including the CH regions) were amplified using primers. The primers that were used anneal to the flanking regions of the VHH coding sequence and are, therefore, not relevant to the selected VHHs. The primer sequences used in the production of the VHH libraries are part of the intellectual property of QVQ Holding BV. The samples were loaded on a 1% TAE agarose gel and the 700 bp fragment (corresponding to VHH encoding cDNA) was excised from the gel and purified. The sample was amplified using nested PCR in which primers were used that introduce SfiI and BstEII restriction sites. The ends of the PCR amplicons were digested with restriction enzyme (Sfil and BstEII) and the sample was loaded on a 1.5% TAE agarose gel. The resulting 400 bp fragment was excised from the gel and purified. The purified 400 bp fragments were ligated into the phagemid pUR8100 vector and transformed into *E. coli* TG1. The transformed TG1 were titrated using 10-fold dilutions. A total of 5 μL of the dilutions was spotted on LB-agar plates supplemented with 100 μg/mL of ampicillin and 2% glucose to determine the library size. Phages were produced from the library and the number of phages per ml (phage titre) was determined. For the 1st round of selections, 20 μL of the precipitated phages was pre-blocked and applied to wells coated with PDGFRB in varying concentrations of 0–0.5–5 μg/mL. TG1 cultures infected with the output of the selection on 5 μg/mL of PDGFRB were used for phage production, in order to perform the 2nd round of selection. For the 2nd round of selection, 1 μL of the precipitated phages was applied to wells coated with PDGFRB in varying concentrations of 0–0.05–0.5–5 μg/mL, where the lowest concentration should result in the highest affinity binders. The high affinity binders from the phage library were used to infect *E. coli* TG1. The cultures were plated out to pick single clones. To test the single clones, periplasmic extracts containing monoclonal VHH were produced and tested using an ELISA assay. A subset of binding monoclonal VHH were sequenced and picked for further analysis. After selection of the final clones, they were re-cloned to yeast expression vector pyQVQ11. This resulted in the production of VHH with the C-direct tag containing a cysteine residue for directional labelling with either biotin, HighLight488 (HL488) or IRDye800cw (LICOR).

### 2.5. Binding of Biotin-Labelled VHH to Purified PDGFRB

Nunc Maxisorp 96-well plates were coated with 2 µg/mL of human PDGFRB extracellular domain (ECD) (His & Fc tag, Sino Biological or 4 µg/mL of murine PDGFRB ECD (R&D Systems), 100 µL per well, at 4 °C overnight. The wells were washed three times with PBS and blocked in PBS/4% BSA for one hour at RT. The wells were washed another three times before incubation with VHH-biotin diluted at 0.01–1000 nM for one hour at RT. The wells were washed six times with PBS, after which bound VHH was detected with o-phenylenediamine dihydrochloride (OPD) 0.4 mg/mL in 0.05 M of phosphate-citrate buffer pH 5.0, with 0.03% sodium perborate (Sigma-Aldrich, St. Louis, MO, USA), 100 µL per well for 30 min at RT in the dark. After 30 min, the reaction was stopped with 50 µL of 1.5 M HCl and optical density was measured at 492 nm on a Synergy H1 microplate reader (BioTek, Santa Clara, CA, USA). Absorbance from non-specific binding in the absence of PDGFRB ECD was subtracted from the absorbance signal from wells with PDGFRB ECD present.

### 2.6. Binding of HL488-Labeled VHH to PDGFRB Expressing HEK293T Cells 

HEK293T cells stably expressing PDGFRB (HEK293T-PDGFRB) were obtained from Zealand Pharma, Denmark. The cells were cultured in DMEM high glucose supplemented with 10% FCS, 1% sodium pyruvate, 1% non-essential amino acids, 1% L-glutamine, 1% penicillin/streptomycin and 300 µg/mL of hygromycin. HEK293T cells were purchased from ECACC/Sigma Aldrich and cultured in DMEM high glucose with 10% FCS, 1% L-glutamine and 1% penicillin/streptomycin. Cells grown in flasks were detached by incubation with trypsin, counted and resuspended in PBS/10% FCS. Each sample contained 200,000 cells. The cells were incubated with VHH for one hour at 37 °C. After washing the cells two times in PBS/2% FCS/5 mM EDTA, PI was added to the buffer in a 0.1 µg/mL concentration as a live/dead stain. Flowcytometry measurements were performed using the MacsQuant from Miltenyi Biotec; data analyses were carried out using FlowJo version 10.

### 2.7. Organoid Culture

Human colorectal cancer organoid line TOR10 was cultured as previously described [23]. In brief, organoids were grown in Matrigel (Corning) and passaged once a week using Trypsin. Organoids were grown in CRC organoid medium (Advanced-DMEM/F12 supplemented with Pen/Strep 50 units/mL, HEPES 10 mM, Glutamax 2 mM, B27 1X, noggin ~100 ng/mL, A38-01 0.5 µM, SB202190 10 µM and *N*-acetylcysteine 1.25 mM). For the intraperitoneal injection of the organoids, one-day-old organoids were harvested using Dispase (1 mg per ml of medium), then counted and diluted in PBS.

### 2.8. Pharmacokinetic In Vivo Analysis

To assess the pharmacokinetics of VHH-IRDye800cw, adult male C57Bl/6 mice (*n* = 3, weight approx. 25–30 g) were injected with VHH-IRDye800cw in the tail vein. The administered dose was 40 µg in 100 µL of PBS. Blood was drawn from the cheek vein at 5 min, 20 min and 60 min post injection. Blood samples were collected in K3EDTA tubes, centrifuged at 2000× *g* for 5 min at 4 °C, and plasma was extracted and stored at −80 °C until analysis. VHH concentration was measured using a direct fluorescence assay and an ELISA measurement. For the direct fluorescence assay, 18 µL samples were measured in 384 well black plates at 750/791 nm using the Synergy H1 plate reader (Biotek). The standard curves of VHH2-IRDye800cw were prepared in PBS and measured in triplicate on the same plate. For the ELISA measurement, Nunc Maxisorp 96-well plates were coated with 2 µg/mL of human PDGFRB extracellular domain (ECD) (His & Fc tag, Sino Biological, Eschborn, Germany). Plasma samples were diluted in PBS/1% BSA/0.05% Tween and measured in duplicate. The VHH-IRDye800cw standard curve was measured in triplicate. The samples were incubated for one hour at RT and washed three times. Polyclonal rabbit anti-VHH antibody (QVQ cat. No QE19) was diluted 1:4000 and incubated for one hour at RT, and the wells were washed three times. Donkey anti-rabbit-HRP (Jackson ImmunoResearch, 711-035-152) was diluted 1:5000 and incubated for one hour at RT, and the wells were washed three times. Bound VHH-IRDye800cw was detected with o-phenylenediamine dihydrochloride (OPD) 0.4 mg/mL in 0.05 M of phosphate-citrate buffer pH 5.0 with 0.03% sodium perborate (Sigma-Aldrich), for 30 min at RT in the dark. After 30 min, the reaction was stopped with 1.5 M of HCl, and optical density was measured at 492 nm on a Synergy H1 microplate reader (BioTek). Fluorescence from blank wells was subtracted from the standard curve and sample values.

### 2.9. In Vivo Biodistribution and Tumour Binding of VHH-IRDye800cw

To study the in vivo biodistribution and tumour binding of VHH-IRDye800cw, 20-week-old male NSG mice (*n* = 8, Charles River) were injected with 500.000 single cells derived from human colorectal cancer organoids (TOR10) into the peritoneal cavity. This resulted in the development of peritoneal metastasis during the course of 8–10 weeks. When the mice showed any signs of discomfort or a stabilizing/decreasing bodyweight, we proceeded with tracer injection, termination and scanning of the organs. The mice were injected with 100 µg (approx. 6.5 nmol) of VHH-IRDye800cw or a nanobody targeting HIV as a negative control, diluted in 100 µL of PBS, in the tail vein. Four hours after injection, the mice were anesthetized using isoflurane gas and euthanized through exsanguination via a cardiac puncture. The collected blood, organs (kidney, muscle, skin, peritoneum, spleen and liver) and peritoneal metastasis were scanned ex vivo in the Pearl Small Animal Imager (LI-COR). The tissues were scanned using 170 µm resolution and 800 nm laser settings. After scanning, the tissues were embedded in OCT and snap frozen. The IRDye800cw signal in the tissues was quantified using the Pearl Cam Software. Same sized regions of interest were drawn, and total fluorescence measurements were used. To visualize the VHH/negative control distribution within tissue sections, peritoneal metastasis was sectioned on a cryostat and fixated with 4% paraformaldehyde for 15 min at RT. The sections were covered in PBS to prevent the tissues from dehydrating. The tumour sections were scanned in the Amersham Typhoon Biomolecular Imager (Cytiva, Marlborough, MA, USA) to detect the IRDye800cw signal. Images were obtained using the IRlong settings (Ex 785 nm, Em 810–840 nm) at a 10 µm pixel size and slow scan speed. Quantification of the IRDye800cw signal from the tumour sections was carried out using ImageQuant. A region of interest was drawn along the borders of the peritoneal metastasis and the mean fluorescence intensity was used.

### 2.10. Immunohistochemistry

Immunohistochemistry was performed on human formalin-fixed paraffin-embedded (FFPE) sections and murine cryosections. Human FFPE tissues were obtained from the same patient cohort that was used for the RNA sequencing analysis of peritoneal metastasis (GSE190609). The FFPE sections (4 µm) were deparaffinized in xylene and rehydrated using a series of alcohol solutions. The sections were blocked using a 1.5% hydrogen peroxide solution for 20 min. For antigen retrieval, the sections were boiled in an EDTA buffer (pH 9), after which they were incubated with a rabbit anti-human PDGFRB antibody (Cell Signaling Technology 3169, Danvers, MA, USA, 1:50) overnight. After incubation with a poly-HRP-labelled goat anti-rabbit secondary antibody (Immunologic VWRKDPVR110HRP) for 30 min at room temperature (RT), the sections were developed with DAB chromogen and counterstained with haematoxylin. Murine frozen tissues were sectioned on a cryostat (7 µm) and fixated with 4% paraformaldehyde (Sigma-Aldrich 158127) for 15 min at RT. The sections were first blocked with a 1.5% hydrogen peroxide solution for 15 min and next with 5% normal goat serum and 0.3% Triton X-100 for one hour at RT. Cryosections were incubated with a rabbit anti-PDGFRB antibody (Cell Signaling Technology 4564, 1:200) and goat-anti-rabbit secondary antibody (Immunologic VWRKDPVR110HRP) for 30 min at RT. The sections were developed with DAB and counterstained with haematoxylin. 

### 2.11. Statistical Analysis

Statistical analyses were conducted in R version 4.0.5 for Windows. To assess the relation between PDGFRB expression and the CMS4 identifying gene set, a Pearson’s correlation coefficient was calculated. To calculate survival curves the Kaplan–Meier method was used and tested for significant differences using the log-rank test. PDGFRB expression was compared between the TGCA dataset, primary tumours with peritoneal involvement and the peritoneal metastasis dataset using an ANOVA and Tukey post-hoc analysis. Dose–response curves and EC50 values were calculated in GraphPad using a nonlinear regression model with variable slope (four parameters). The absorbance values of wells incubated with 0 nM of VHH were included as 10^−12^. The VHH half-life was calculated in GraphPad using a one phase decay nonlinear regression model.

## 3. Results

### 3.1. PDGFRB Expression Identifies CMS4 CRC with High Specificity and Sensitivity

**Figure 1:**
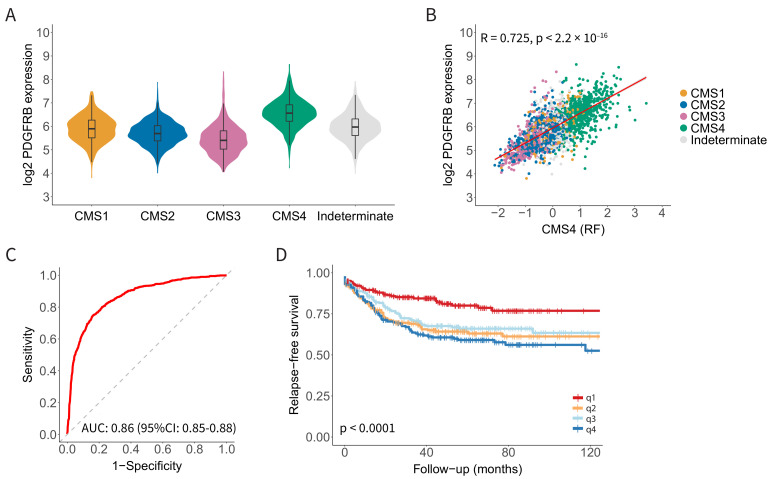
Platelet-Derived Growth Factor Receptor beta (PDGFRB) mRNA expression identifies Consensus Molecular Subtype 4 (CMS4) colorectal cancer (CRC) with high specificity and sensitivity. (**A**) Violin plot showing PDGFRB expression in the CMS subgroups in a composite RNA sequencing cohort of 3232 primary CRC tumours. (**B**) Scatter plot showing a strong correlation between PDGFRB expression and the CMS4-identifying gene set (*n*= 143 genes; CMS4 (RF)) from the CMS classifier in the composite CMS-3232 cohort. (**C**) Receiver operating characteristic (ROC) curves showing the sensitivity and specificity of using PDGFRB mRNA levels to distinguish between CMS4 and CMS1-3. The Area Under the Curve (AUC) value is shown, as a measure of the diagnostic accuracy of the PDGFRB test. (**D**) Kaplan–Meier curves displaying relapse-free survival probabilities in subgroups defined by PDGFRB expression quartiles, *n* = 805 patients. There is a significantly worse relapse-free survival probability in the higher PDGFRB expression quartiles.

We recently performed a differential gene expression analysis to identify candidate targets for molecular imaging that would allow the distinction between ‘mesenchymal-like’ CMS4 tumours and ‘epithelial-like’ CMS2 tumours [15]. This effort identified FAP and PDGFRB as candidate targets for the molecular imaging of CMS4 CRC [15]. The expression of PDGFRB was significantly higher in CMS4 than in CMS1-3 (Figure 1A). Furthermore, PDGFRB expression correlated very well with the expression of the CMS4-identifying gene-set from the CMS random forest classifier (R = 0.72; *p* < 2.2 × 10^−16^; Figure 1B). As a single gene, the expression of PDGFRB performed remarkably well in predicting CMS4 status in the original composite cohort of 3232 primary tumours [21] (AUROC = 0.86; 95% CI: 0.85−0.88) (Figure 1C). Finally, the high expression of PDGFRB was significantly correlated with an increased chance of distant relapse (Figure 1D). Taken together, the data indicate that PDGFRB is an excellent single-gene identifier of the relapse-prone CMS4 CRC.

### 3.2. High and Uniform Expression of PDGFRB in CMS4 Peritoneal Metastases

Virtually all peritoneal metastases from CRC belong to CMS4 [17,18,19]. Therefore, we analysed PDGFRB mRNA and protein levels in the primary CRC tumours of the TCGA cohort (*n* = 582; without known peritoneal involvement); in primary tumours with peritoneal involvement (*n* = 35 regions from 12 patients); and in paired peritoneal metastases (*n* = 59).

**Figure 2:**
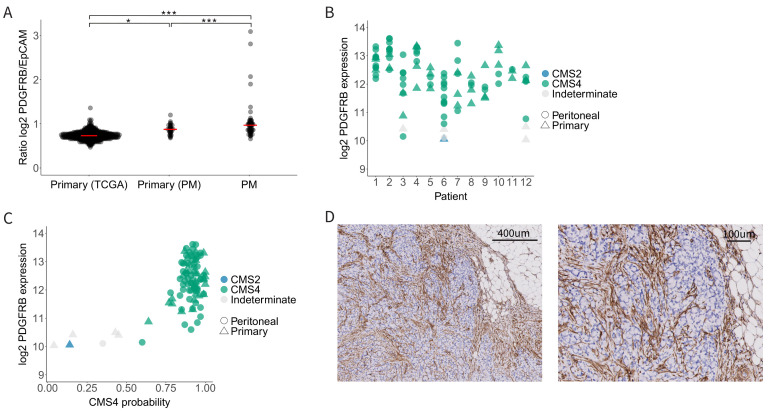
PDGFRB expression in CMS4 peritoneal metastases. (**A**) Relative PDGFRB expression levels in the TCGA cohort (primary CRC tumours, *n* = 592) versus the peritoneal metastasis cohort (primary tumours with peritoneal dissemination, *n* = 35, and the peritoneal metastases derived from them, *n* = 59). Each dot represents a tumour sample, the red bar represents the median PDGFRB/EpCAM ratio. (**B**) Dot plot showing PDGFRB expression levels in primary tumours and their matched peritoneal metastases. Tissue type (primary vs. PM) is annotated by shape and CMS classification is color-coded. (**C**) Scatter plot showing the correlation of CMS4 probability with PDGFRB mRNA levels in the peritoneal metastasis cohort. PDGFRB mRNA levels correlate very well with CMS4 probability of the same lesions. (**D**) Immunohistochemistry for PDGFRB on peritoneal metastasis tissue from the same peritoneal metastasis cohort. The tumour stroma of 17/18 peritoneal metastasis was positive for PDGFRB. Significance levels * *p* < 0.05, *** *p* < 0.001.

The highest levels of PDGFRB mRNA expression were observed in peritoneal metastases (Figure 2A). Furthermore, primary tumours with peritoneal involvement expressed higher levels of PDGFRB than those without peritoneal involvement (Figure 2A). PDGFRB expression was generally very high among all peritoneal metastases, but it was still variable (Range 2log expression values 10–14) (Figure 2B) and correlated very well with CMS4 probability (Figure 2C). Indeed, the few tumours and metastases that did not classify as CMS4 also had the lowest expression of PDGFRB (Figure 2C). Next, we performed immunohistochemistry to analyse the PDGFRB protein expression in 18 peritoneal metastases from 11 individual patients. This revealed strong PDGFRB staining in 94% (17/18) of the peritoneal metastases, compared to the surrounding normal tissue (Figure 2D and Supplemental Figure S1). We conclude that CMS4 peritoneal metastases uniformly express high levels of PDGFRB mRNA and protein in the stromal fibroblasts. 

### 3.3. Generation of a PDGFRB-Binding Nanobody (VHH1E12)

**Figure 3:**
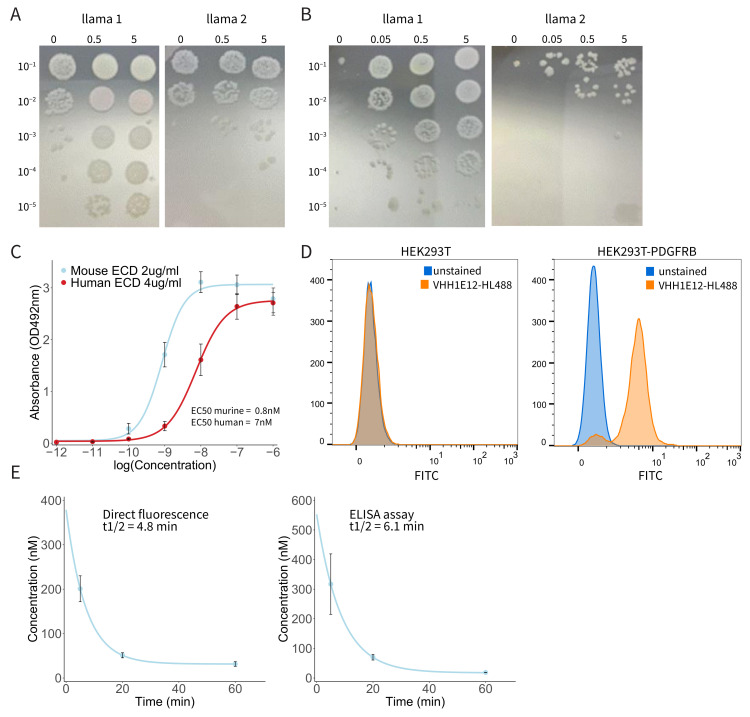
PDGFRB-binding nanobody VHH1E12 binds with nanomolar affinity to human and murine PDGFRB. (**A**) First round of phage selection. Precipitated phages were applied to wells coated with a range of PDGFRB concentrations. Outputs of the binding phages from wells coated with 5 μg/mL of PDGFRB were eluted and used to infect new TG1 cultures. (**B**) Second round of phage selection. Outputs of phages binding to wells coated with 0.05 μg/mL of PDGFRB were eluted from the wells and used to infect new TG1 cultures. Outputs from the first and second round of phage selections were cultured for single clone picking, after which monoclonal VHHs were selected for further analysis. (**C**) Dose–response curve of biotin labelled VVH1E12 binding to human and murine Extra Cellular Domain (ECD) of PDGFRB. Mean absorbance with SEM is shown from four independent experiments. EC50 human PDGFRB = 7 nM; EC50 murine PDGFRB = 0.8 nM. (**D**) Dose response of HighLight488-conjugated VHH1E12 to HEK293T-PDGFRB cells. The left graph shows the percentage of cells positive for VHH1E12 and the right graph shows the MFI. (**E**) Pharmacokinetic analysis of IRDye800cw labelled VHH1E12. Plasma levels were assessed by direct fluorescence measurement (left graph) and using an anti-VHH secondary antibody and ELISA assay (right graph). Mean of three animals with SEM is shown.

Having validated PDGFRB as an attractive target for the detection of CMS4 CRC, particularly peritoneal metastases, we next decided to generate a PDGFRB-targeting nanobody for application as a molecular imaging tracer. Nanobodies are small (~15 kDa) single-domain camelid VHH antibody fragments. They are ideally suited for molecular imaging purposes because of their potential to bind target molecules with very high specificity and affinity, their rapid clearance from the circulation, and their tissue penetration capacity [24]. After the immunization of two llamas with PDGFRB, RNA from PBMCs was reverse-transcribed into cDNA. Fragments that encode VHH were amplified using specific VHH primers and incorporated in *E. coli* TG1. To determine the phage library size, TG1 cultures were diluted and spotted on LB-agar plates. Both phage libraries were of good size with >10^7^ clones per library. Phage titres were determined and were both >10^11^ phages/mL, corresponding to >1000 fold the diversity of the libraries. For the first round of phage selection, 20 μL of the precipitated phages was applied to wells coated with a range of PDGFRB concentrations (Figure 3A). Outputs of the binding phages from wells coated with 5 μg/mL of PDGFRB were eluted and used to infect new TG1 cultures. They were used for phage production for the second round of selection. In this second selection round, 1 μL of precipitated phages was applied to wells with PDGFRB (Figure 3B). There was a clear concentration-dependent enrichment between the different PDGFRB concentrations, indicating that the selected VHH bind specifically to PDGFRB. Phages binding to wells coated with 0.05 μg/mL should result in the highest affinity binders. Outputs were eluted from the coated wells and used to infect new TG1 cultures. Subsequently, outputs of the first and second round of selection were plated out in order to pick single clones. A total of 92 single clones were picked in a 96 wells plate. Periplasmic extracts containing monoclonal VHH were produced and tested for specificity using an ELISA assay. Based on the ELISA, a total of nine clones were selected for sequencing. The sequence alignment shows a diversity of at least five different VHH sequences based on the epitope family. A total of four clones were picked for further analysis. Within these four clones there was one that showed binding affinity to both human and murine PDGFRB. With this clone, VHH1E12, we continued our research.

### 3.4. Binding of Biotin-Labelled VHH1E12 to Purified PDGFRB

As seen in the immunohistochemistry staining of PDGFRB in peritoneal metastases, PDGFRB expression is mainly localized in the tumour stroma. In particular, Cancer Associated Fibroblasts (CAFs) show a strong PDGFRB expression. In patient-derived organoid xenograft models the tumour consists of human cancer cells, but the tumour stroma is formed by murine cells. It is, therefore, important for in vivo evaluation to use a VHH that has a high affinity for both murine and human PDGFRB. To test this, we used an ELISA assay with plates coated with human and murine PDGFRB Extra Cellular Domain (ECD), which were incubated with a range of VHH concentrations. Dose–response curves were calculated from four independent experiments (Figure 3C). VHH1E12 showed an EC50 of 7 nM for human and 0.8 nM for murine PDGFRB, making it a promising candidate to test PDGFRB binding in vivo. 

### 3.5. Binding of HL488-Labeled VHH1E12 to Cell Surface-Expressed PDGFRB

Next, we were interested in the ability of VHH1E12 to bind PDGFRB on live cells. For this we used HEK293T cells and a HEK293T cell line transduced with human PDGFRB. Both cell lines were incubated with a VHH1E12-HighLight488 (HL488) conjugate. After a one-hour incubation period with 10 nM of VHH1E12 at 4 °C, 90.5% of the HEK293T-PDGFRB cells were positive for VHH1E12-HL488 (Figure 3D and Supplemental Figure S2). The naïve HEK293T cell line showed no binding of VHH1E12. 

### 3.6. Pharmacokinetic Analysis of IRDye-800CW-Conjugated VHH1E12 in Mice

Nanobodies are known for having a short in vivo half-life. All proteins smaller than 65 kDa are below the renal threshold for first-pass clearance. In general, monomeric nanobodies (~15 kDa) have a half-life between 30 min and two hours. We tested the blood plasma half-life in three mice using two quantification methods. Using an assay in which the direct fluorescence of IRDye800cw was detected in the blood plasma, the half-life was 4.8 min. The half-life detected through the binding of a secondary anti-VHH antibody in an ELISA assay was 6.1 min (Figure 3E). 

### 3.7. Detection of PDGFRB in Experimental Peritoneal Metastases by IRDye-800CW-Conjugated VHH1E12

**Figure 4:**
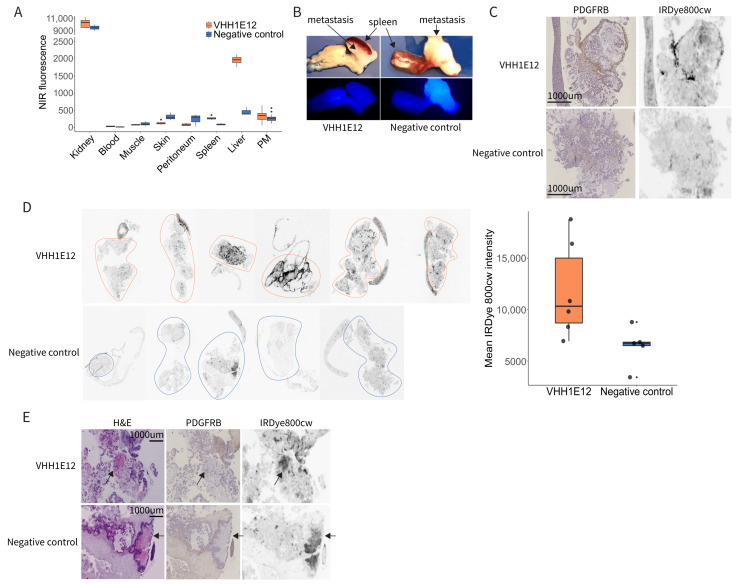
Detection of PDGFRB in experimental peritoneal metastases by IRDye-800CW-conjugated VHH1E12. (**A**) Quantification of near-infrared (NIR) signal of VHH1E12 and negative control in kidney, blood, muscle, skin, peritoneum, liver and peritoneal metastasis (PM). Whole organs and PMs were scanned in the Pearl NIR small animal imager. A same sized region of interest (ROI) was drawn in all the scanned organs and total fluorescence intensity was used. VHH1E12 *n* = 4 mice; negative control *n* = 3 mice. (**B**) Representative NIR scan of peritoneal metastasis uptake between VHH1E12 and the negative control on a whole tumour level. (**C**) PM tissue sections stained for PDGFRB using immunohistochemistry and scanned for IRDye800cw signal in the Typhoon scanner. (**D**) Left panel: PM tissue sections scanned for IRDye800cw signal in the Typhoon scanner. VHH1E12 *n* = 4 mice and *n* = 6 PMs; negative control *n* = 3 mice and *n* = 5 PMs. ROIs are drawn around the tumour tissue. Right panel: quantification of IRDye800cw in PM tissue sections. Mean pixel intensity is plotted for VHH1E12 *n* = 6 PM and negative control *n* = 5 PM. (**E**) Sequential PM tissue sections stained with haematoxylin and eosin showing areas of necrosis with strong eosin staining, indicated by the black arrows. These areas were negative for PDGFRB and showed a strong IRDye800cw signal.

Tumour accumulation and the biodistribution of VHH1E12-IRDye-800cw was assessed by imaging the near-infrared (NIR) signal in a mouse xenograft model of peritoneal metastases. Four hours after the intravenous injection of either VHH1E12 or the anti-HIV nanobody used as a negative control, the mice were euthanized, and organs and peritoneal metastases were imaged ex vivo in the Pearl Imager. Looking at the biodistribution of both compounds in healthy tissue, there was a significantly higher uptake of VHH1E12 in the liver (Figure 4A). Cryosections of liver tissue showed a diffuse uptake of VHH1E12 and almost no PDGFRB expression, making it unlikely that the uptake in the liver of VHH1E12 is caused by specific binding to PDGFRB. 

On a whole tumour level there was a small, non-significant specific uptake of VHH1E12 in peritoneal metastasis when compared to the negative control (Figure 4B). To assess the specificity of tracer accumulation, tumour cryosections were stained for PDGFRB. The tumour stroma of peritoneal metastasis clearly showed PDGFRB expression. (Figure 4C). Sequential cryosections were used to assess VHH1E12 and negative control uptake on a tissue section level. Peritoneal metastasis from mice injected with VHH1E12 showed on average a 1.7-times higher NIR signal compared to the negative control. Furthermore, the nanobody signal co-localized with PDGFRB expression (Figure 4C,D), indicating a specific binding of VHH1E12 to the PDGFRB receptor in vivo. Some areas on the tumour cryosections showed a strong NIR signal without the presence of PDGFRB. These NIR positive areas resembled necrotic tissue on the PDGFRB IHC slide, which was confirmed by a strong eosin staining on the H&E section (Figure 4E). We saw these NIR positive necrotic areas in tumours from mice injected with VHH1E12 and the negative control, suggesting that the binding to necrotic areas is caused by the non-specific binding of the IRDye800cw fluorescent tag. 

## 4. Discussion

This report validates PDGFRB as an attractive target for the detection of CMS4 CRC, and in particular peritoneal metastases, through molecular imaging with a PDGFRB-targeting nanobody. Recent work has demonstrated the potential value of FAPI-PET imaging in the detection of peritoneal metastases [14,15,16]. The completely different tracer backbones (i.e., a small molecule inhibitor versus a nanobody) suggests that both tracer types may have their advantages and disadvantages in various clinical applications. The need for new imaging modalities to improve peritoneal metastasis detection is strong. A PDGFRB-targeting molecular imaging strategy for peritoneal metastasis detection has multiple potential clinical applications. First, it is a non-invasive method that allows for long-term follow-up in patient groups at risk for developing peritoneal metastasis. Second, it can be used to evaluate the response of peritoneal metastasis to systemic therapy. It is currently difficult to evaluate treatment response in patients with peritoneal metastasis receiving systemic therapies, due to the lack of effective imaging modalities. As the symptoms caused by peritoneal metastasis growth overlap with the toxic side-effects of systemic therapy it is often difficult to distinguish disease progression from treatment-related toxicity. Lastly, it can help to improve patient selection for cytoreductive surgery and hyperthermic intraperitoneal chemotherapy, including elderly patients (HIPEC) [25,26]. In the present study, we demonstrate the specific uptake of a PDGFRB-targeting nanobody, VHH1E12, in peritoneal metastases in mice. A clinical pilot study is now needed to assess whether VHH1E12 (or similar nanobodies) can be used to detect peritoneal metastases in cancer patients.

Theoretically, PDGFRB-binding tracers could influence PDGFR signalling in the tumour microenvironment. PDGFRB signalling mainly has pro-tumorigenic and pro-metastatic effects [27]. The activation of PDGFRB on tumour cells stimulates proliferation, invasion and can induce epithelial-to-mesenchymal transition (EMT). Consequently, PDGFRB expression in mesenchymal colorectal tumour cell lines promotes the formation of liver metastases [28]. In addition, the activation of PDGFRB on stromal fibroblasts also has pro-tumorigenic effects [27,29]. The collagen-rich extracellular matrix in peritoneal metastases has the potential to activate platelets, which connects their stroma-richness to an aggressive PDGF-ligand-rich tumour phenotype. While nanobodies bind PDGFRB with high affinity, the administered tracer dosages are usually very low, and the half-lives are short. Therefore, we deem it unlikely that tracers such as VHH1E12 would have a major impact on PDGFRB signalling in the tumour microenvironment.

The VHH1E12 nanobody generated in this study showed non-specific retention in the liver, thereby hampering its application in the detection of liver metastases. The only PDGFRB-expressing cells in the healthy liver are hepatic stellate cells. However, the relatively low abundance of these cells cannot account for the observed homogeneous tracer retention. The most likely explanation is that VHH1E12 is taken up by hepatocytes as part of its metabolism, and that this is different from the metabolism of the negative control nanobody. In addition, we found that both the PDGFRB-targeting and control nanobodies accumulated in areas of tumour necrosis. Some in vivo studies have shown that near-infrared cyanine dyes such as IRDye800cw have an affinity for tissue necrosis [30,31]. For diagnostic purposes in patients, the IRDye800cw group will be exchanged for a radio isotope, circumventing the issue of non-specific binding to tumour necrosis. 

An in vitro analysis showed that VHH1E12 has a sub-nanomolar affinity for the murine PDGFB-receptor and a nanomolar affinity for the human receptor. This compares favourably to other developed radiotracers in the field. However, the short in vivo half-life (6 min) was unexpected based on the size of the nanobody (15 kDa). This short circulation time could hamper the necessary binding of VHH1E12 to the PDGFB-receptor for optimal diagnostics. There are several methods to prolong the half-life of nanobodies, e.g., by fusion to an albumin-binding nanobody or PEGylation [32]. Additional modifications to the PDGFRB-targeting nanobody may be generated to optimize tumour-specific uptake and retention. 

Affibodies represent an alternative platform for developing PDGFRB-based molecular imaging [33,34]. Affibodies are derived from *Staphylococcus aureus* protein A and are even smaller than nanobodies [35]. Both formats are ideally suited for developing molecular imaging strategies in cancer [24,35]. In addition, radiolabelled small molecule PDGFR-family inhibitors such as imatinib and dasatinib can also be used as molecular imaging tracers [36,37]. Future work should assess the merits and disadvantages of the various PDGFRB-targeting platforms (affibody, nanobody, small molecule, etc.) in terms of pharmacokinetics, binding affinities and specificities, and tumour tissue retention. 

The latter parameter is especially important for potential future theranostic applications, as prolonged tracer retention in the tumour is required to obtain sufficient radiation exposure of the targeted tissue. In the case of peritoneal metastases from CRC, PDGFRB-targeting tracers could be used in a ‘targeted endo-radiation’ approach. This could offer an urgently needed alternative treatment strategy against this therapy-refractory disease entity. Diagnostic or therapeutic PDGFRB-targeting tracers may be applied systemically or inside the peritoneal cavity. A potential advantage of the latter approach is that systemic toxicity is likely to be very limited in therapeutic applications. 

An additional potential future application is the use of fluorescent tracers in imaging-guided surgery. Proof-of-concept for the potential value of the imaging-guided surgery of peritoneal metastases has already been provided using antibody-based tracers recognizing carcinoembryonic antigen (CEA) or vascular endothelial growth factor A (VEGFA). Both tracers revealed the presence of previously undetected lesions [38,39]. 

## 5. Conclusions

We conclude that PDGFRB is an attractive target for the molecular imaging of CMS4 CRC, including peritoneal metastases. Future work should include a comparison of the performance of the specific tracer formats (nanobody, affibody, small molecule inhibitor) in the various foreseen clinical applications. These include the quantitative whole-body diagnosis of CMS4, assessment of the extent of intraperitoneal disease, intra-operative imaging, and PDGFRB-targeted endo-radiotherapy. Finally, PDGFRB-targeted diagnostic and therapeutic approaches are likely to be valuable in additional tumour types such gastrointestinal stromal tumours (GIST) and stroma-rich carcinomas such as pancreatic cancer. 

## Data Availability

All used RNA sequencing datasets are publicly available; see methods section colorectal cancer RNA sequencing datasets for corresponding repositories.

## Supplementary Materials

The following supporting information can be downloaded at: https://www.mdpi.com/article/10.3390/cancers14184348/s1, Figure S1: PDGFRB immunohistochemistry on 17 peritoneal metastases from 12 patients; Figure S2: Flow cytometry gating strategy of HEK293T-PDGFRB cells.
