# Supplementary material for: Detection of Experimental Colorectal Peritoneal Metastases by a Novel PDGFRβ-Targeting Nanobody
Source: Cancers (Basel). 2022 Sep 6;14(18):4348. doi: 10.3390/cancers14184348 (PMC9497196; doi:10.3390/cancers14184348)
Supplement: Supplementary file 1 [file cancers-14-04348-s001.zip › cancers-1834270-supplementary.pdf]

Supplemental figure S1.

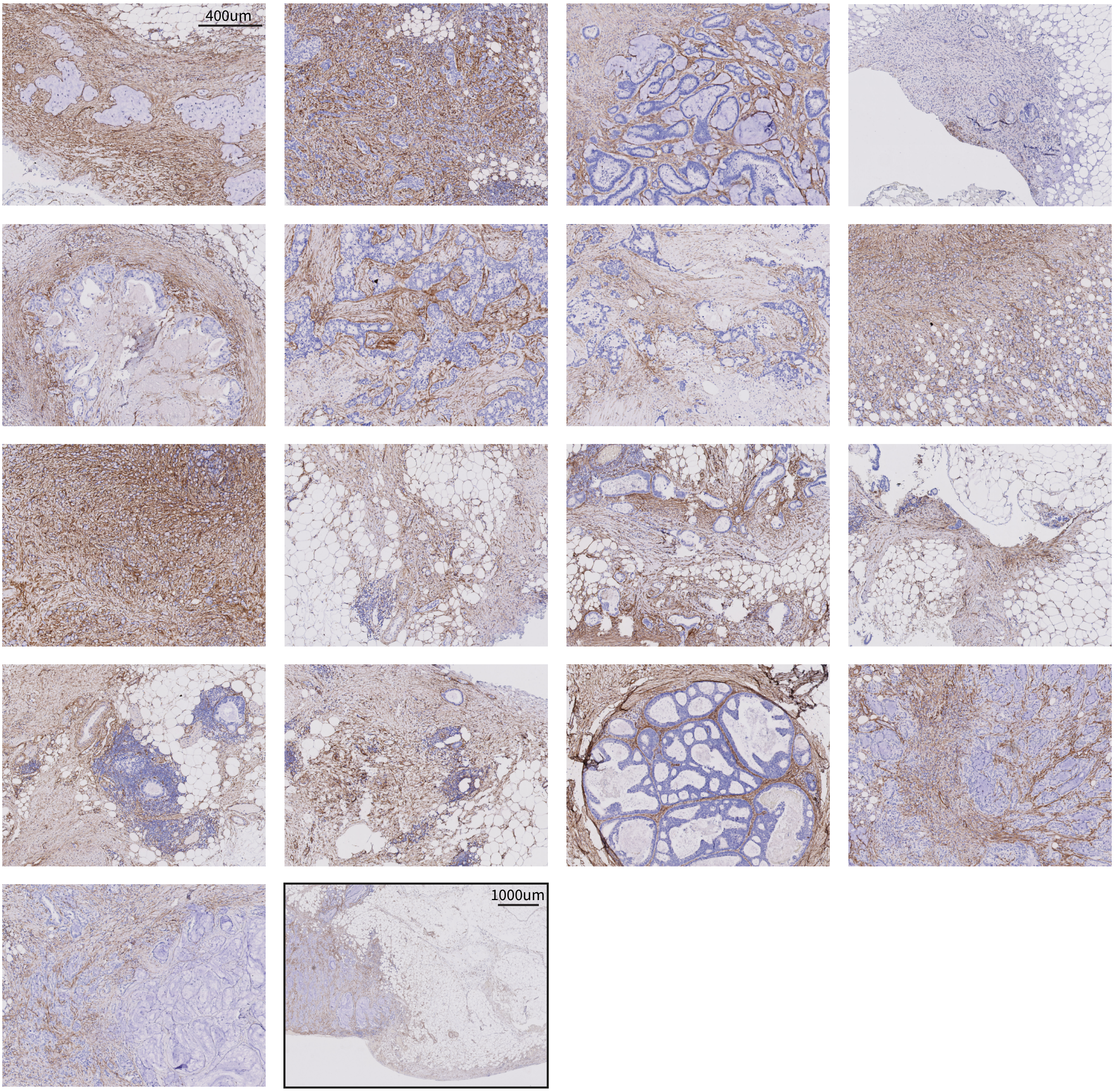

Figure S1. PDGFRB immunohistochemistry on 17 peritoneal metastases from 12 patients. Peritoneal metastases uniformly express PDGFRB. The last image (lower magnification and black outline) shows PDGFRB expression in tumor adjacent tissue.

Supplemental figure S2.

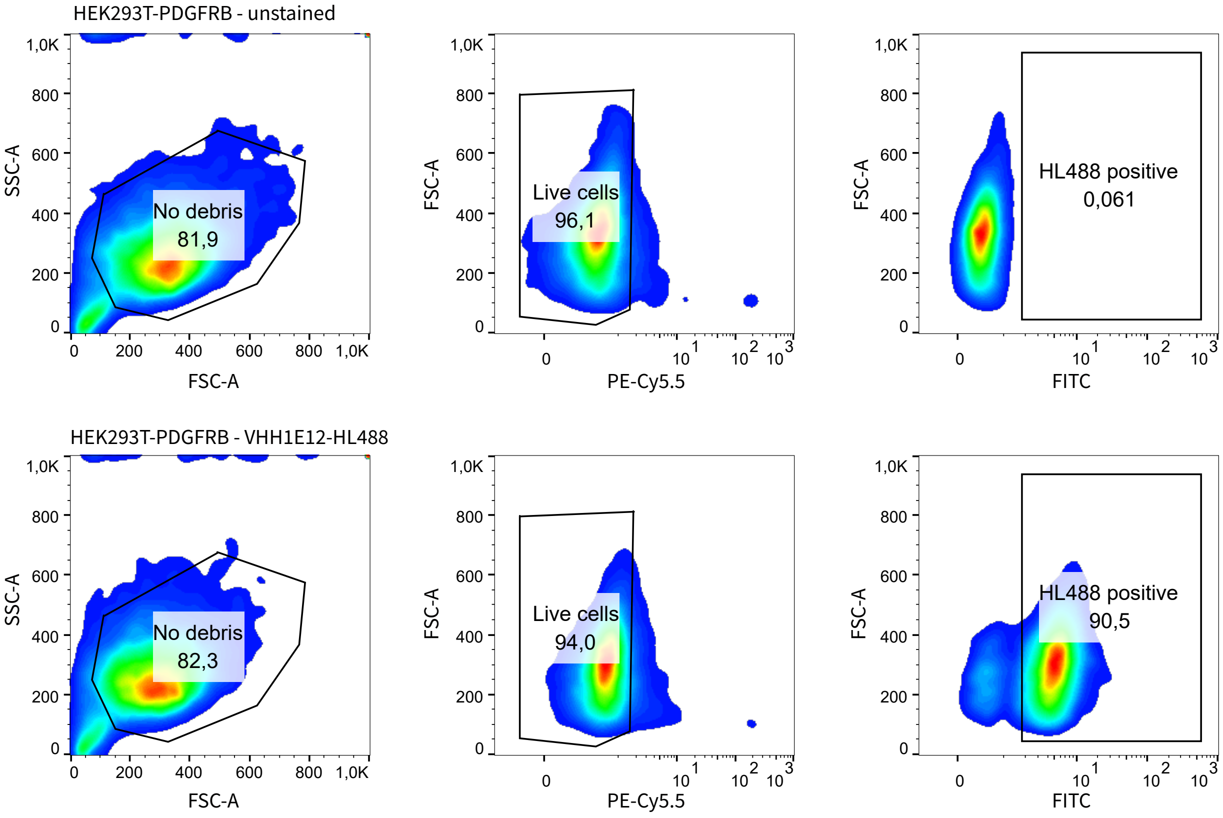

Figure S2. Flowcytometry gating strategy of HEK293T-PDGFRB cells incubated with 10nM VHH1E12-HL488 for 1 hour at 4C compared to the unstained control. Debris is excluded in the first gate in the FSC/SSC plot. In the second plot only cells that are negative for PI (live/dead stain) are selected, in the last plot HL488 positive cells are selected. After incubation with VHH1E12-HL488 90.5% of the cells were positive for HL488.
